# EPA guidance on treatment of cognitive impairment in schizophrenia

**DOI:** 10.1192/j.eurpsy.2023.124

**Published:** 2023-07-19

**Authors:** A. Vita

**Affiliations:** Department of Clinical and Experimental Sciences, University of Brescia, Brescia, Italy

## Abstract

**Abstract:**

Although cognitive impairment is a core symptom of schizophrenia related to poore outcome in different functional domains, it still remains a major therapeutic challenge. To date, no comprehensive treatment guidelines for cognitive impairment in schizophrenia are implemented.

The aim of the European Psychiatric Association (EPA) guidance paper was to provide a comprehensive meta-review of the current available evidence-based treatments for cognitive impairment in schizophrenia, structured into three sections: pharmacological treatment, psychosocial interventions, and somatic treatments.

Based on the reviewed evidence, the EPA guidance recommends an appropriate pharmacological management as a fundamental starting point in the treatment of cognitive symptoms in schizophrenia. Among psychosocial interventions, cognitive remediation and physical exercise are recommended for the treatment of cognitive impairment in schizophrenia. As for cognitive remediation, some variables have been confirmed as core elements for treatment effectiveness.

The dissemination of the EPA guidance paper may promote the development of shared guidelines concerning the treatment of cognitive functions in schizophrenia, with the purpose to improve the quality of care and to achieve clinical recovery in this population.

**Disclosure of Interest:**

None Declared

